# Design and rationale of the North Indian ST‐Segment Elevation Myocardial Infarction Registry: A prospective cohort study

**DOI:** 10.1002/clc.23278

**Published:** 2019-10-08

**Authors:** Sameer Arora, Arman Qamar, Puneet Gupta, Muthiah Vaduganathan, Ishit Chauhan, Ashutosh K. Tripathi, Vinamra Y. Sharma, Ankit Bansal, Amber Fatima, Gagan Jain, Vishal Batra, Sanjay Tyagi, Lokesh Khandelwal, Prashant Kaul, Sunil V. Rao, Meenahalli Palleda Girish, Deepak L. Bhatt, Mohit D. Gupta

**Affiliations:** ^1^ Division of Cardiology University of North Carolina Chapel Hill North Carolina; ^2^ Preventive Medicine Residency, Department of Family Medicine University of North Carolina Chapel Hill North Carolina; ^3^ Brigham and Women's Hospital Heart and Vascular Center, Harvard Medical School Boston Massachusetts; ^4^ Department of Cardiology Janakpuri Superspeciality Hospital New Delhi India; ^5^ Department of Cardiology Gobind Ballabh Pant Institute of Postgraduate Medical Education and Research New Delhi India; ^6^ Department of Medicine Tufts Medical Center Boston Massachusetts; ^7^ Piedmont Heart Institute Atlanta Georgia; ^8^ The Duke Clinical Research Institute Durham North Carolina

**Keywords:** cardiovascular outcomes, myocardial infarction, registry, STEMI

## Abstract

ST‐segment elevation myocardial infarction (STEMI) is associated with increased mortality and morbidity. Although remarkable progress has been made in the management of STEMI in high‐income countries, contemporary data to evaluate processes and outcomes of STEMI care in India is limited. The North Indian ST‐segment elevation myocardial infarction (NORIN STEMI) registry is a prospective cohort study based at government funded and largely free of cost tertiary medical centers in New Delhi, India. These hospitals serve a large proportion of the patients with lower socioeconomic status presenting from multiple states in India, as many centers in these states lack adequate specialized cardiovascular care. The study has been approved by the Institutional Review Boards of each institution and informed consent has been obtained from study participants. The NORIN STEMI registry aims to provide important insights regarding contemporary risk factors profiles, practice patterns, and prognosis in patients with STEMI in an underserved population in North India. These findings may identify opportunities to improve the outcomes of patients with STEMI in India.

**Abbreviations**
CAD: coronary artery disease
CVD: cardiovascular disease
MI: myocardial Infarction
NORIN STEMI: North Indian ST‐segment elevation myocardial infarction registry
PCI: percutaneous coronary intervention
STEMI: ST‐segment elevation myocardial infarction

## 1 INTRODUCTION

Traditionally seen as a disease of high‐income countries, recent trends have shown an increasing incidence and a major shift in burden of cardiovascular disease (CVD) with greater than 75% of the global burden now occurring in low‐ and middle‐income countries.^1^ While substantial progress has been made in lowering mortality from CVD in high‐income countries, morbidity and mortality from CVD in low‐and middle‐income countries including India remain high.^2^, ^3^, ^4^

ST‐segment elevation myocardial infarction (STEMI) is a life‐threatening manifestation of coronary artery disease (CAD) requiring prompt recognition and emergent reperfusion. The proportion of patients with acute coronary syndromes (ACS) presenting with STEMI are also higher in India.^5^ Although there are concerns about increasing incidence of MI in young worldwide,^6^, ^7^, ^8^, ^9^, ^10^, ^11^, ^12^ the mean age of presentation with ACS in India is ~55 years, approximately 10 years younger than reports from high‐income countries.^13^ More than 50% of patients with STEMI in India are of lower socioeconomic status and are unable to afford basic healthcare, let alone out‐of‐pocket costs for primary percutaneous coronary intervention (PCI) and associated medical therapies.^5^, ^14^ Furthermore, the practice patterns for management of STEMI in India suffer from significant limitations including delays and underutilization of reperfusion therapy and lower rates of adherence to evidence‐based medical therapy.^13^ Compounding these factors are the significant inter‐regional disparities in socioeconomic status across India.^14^ Consequently, mortality from STEMI in India is significantly higher than in North America or Europe.^2^

Despite these alarming trends and burden of disease, there is a lack of contemporary data regarding risk factors, clinical characteristics, practice patterns, and outcomes among patients presenting with STEMI in India. The large‐scale data that has previously emerged has largely been from states which score relatively well on indicators of social determinants of health and therefore, may not be truly representative of the demography of India which continues to remain poor.^15^, ^16^ Indeed, select states in North India have among the highest rates of CVD throughout the nation.^17^, ^18^ STEMI represents an ideal initial target condition given its high associated mortality and well‐established evidence‐based processes of care. A prospective study of STEMI in a North Indian population may identify prevalent risk factors that can be modified to mitigate the risk of atherothrombosis and may also recognize gaps in current practice approaches that can be targeted to improve outcomes of STEMI in India.

### 1.1 Study objectives

Our study aimed systematically and prospectively to characterize patients presenting with STEMI in North India with respect to risk factors, clinical profiles, evidence‐based therapies, clinical outcomes, and predictors of adverse cardiovascular events.

## 2 METHODS

**Figure 1:**
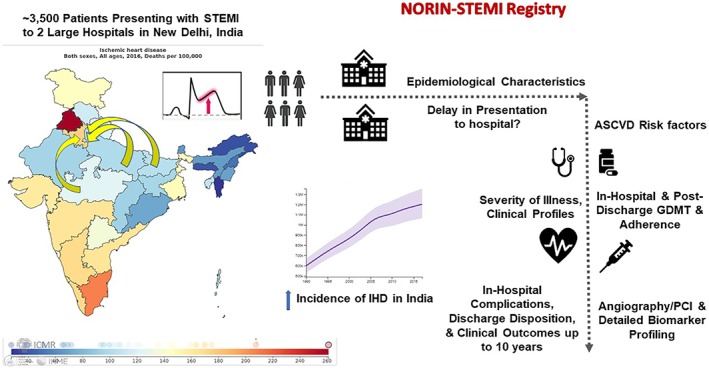
Study schema and key objectives. *Adapted from Global Burden of Disease Results tool and GBD India Compare. NORIN STEMI: North India ST‐Elevation Myocardial Infarction; IHD: ischemic heart disease; ASCVD: atherosclerotic cardiovascular disease; PCI: percutaneous coronary intervention; GDMT: guideline‐directed medical therapy

The NORIN STEMI registry is a prospective observational registry of patients hospitalized with STEMI at two tertiary medical centers in New Delhi, India. This registry started enrolling patients with STEMI in January 2019; Figure 1 illustrates the study schema.

### 2.1 Data source

Gobind Ballabh Pant Institute of Postgraduate Medical Education and Research (GIPMER) and Janakpuri Superspecialty Hospital (JSSH) in New Delhi, India serve as the data source and initial organizations for this registry. GIPMER and JSSH provide care to over 1 million patients every year. These large hospitals located in New Delhi serve a large diaspora of the Indian population with patients presenting from multiple Indian states, including Delhi, Haryana, Punjab, Uttar Pradesh, Madhya Pradesh, Bihar, and North‐Eastern States, as many centers in these states lack adequate PCI capabilities and specialized cardiovascular care. The facilities that exist are largely private funded and require prior payment of the cost of any procedure out‐of‐pocket which is out of financial reach of most patients in these states. In comparison, GIPMER and JSSH are government funded and largely free of cost to the patient and, therefore, are major centers of cardiac care for a large proportion of the North Indian population.^19^, ^20^ GIPMER is one of the largest volume subspecialty‐hospitals all across India with a 24‐hour emergency room, cardiology outpatient department with capabilities for PCI, balloon‐pump, coronary care unit, and backup from cardiothoracic surgery. JSSH is a new Delhi‐Government‐funded subspecialty hospital where recruitment is being done from outpatient department only. Currently, JSSH does not have an emergency department and all patients recruited present to the outpatient department. JSSH is a PCI capable center with a 24‐hour coronary care unit and has balloon‐pump support for hemodynamically unstable patients. Processes are underway to expand into one of the largest subspecialty‐hospital in the West Delhi area with 24‐hour coronary care.

Licensed physicians complete templated electronic questionnaires via a password‐protected smart phone application named NORIN customized for the NORIN STEMI registry. Unique IDs are automatically generated for each new patient. When saved, the data are transmitted to a central server, which can only be accessible by the study investigators. The study protocol has been approved by the Institutional Review Boards of both study sites and informed consent will be obtained from study participants.

### 2.2 Identification of study population and patient selection

Since 1 January 2019, all patients >18 years of age presenting with STEMI and consenting to be a part of the registry are being prospectively enrolled. Written consents will be obtained from all eligible patients. In post‐arrest or intubated patients, the next of kin or the immediate first‐degree relative will provide the consent. Diagnostic ST‐segment elevation in the absence of left ventricular hypertrophy or left bundle branch block will be defined by the European Society of Cardiology/American College of Cardiology Foundation/American Heart Association/World Heart Federation Task Force for the fourth Universal Definition of Myocardial Infarction as new ST‐segment elevation at the J point in at least two contiguous leads of ≥2 mm (0.2 mV) in men or ≥1.5 mm (0.15 mV) in women in leads V2‐V3 and/or of ≥1 mm (0.1 mV) in other contiguous chest leads or the limb leads.^21^ Patients presenting more than 21 days after onset of symptoms were excluded.

### 2.3 Ascertainment of clinical data

A detailed data collection form approved by the Institutional Review Board will be used with brief summary reported in Table S1. Furthermore, each patient will be followed for occurrence of clinical endpoints and adherence to medical therapy. The study organization structure is detailed in online Appendix.

### 2.4 Demographics

Besides baseline demographics as in Table S1, other data points include information on whether patient presented directly to the facility or was transferred; mode of presentation to the hospital (brought in by self or family, ambulance, or public transport), education status, occupational status, dietary habits, physical activity, sleep duration, socioeconomic status, and insurance status. Family history of CAD will be defined as any first‐degree relative with a history of MI or coronary revascularization. Family history of premature CAD will be defined as MI occurring before the age of 55 years for men and before the age of 65 years for women.

### 2.5 Risk factors

All patients will be evaluated for the presence of both traditional and emerging cardiovascular risk factors. Detailed information will be ascertained through patient interviews and by review of medical records. Risk factors will be defined using the current guideline recommendations. Hypertension will be defined as systolic blood pressure ≥140 mmHg, diastolic blood pressure ≥90 mmHg, or diagnosis/treatment of hypertension.^22^ Height and weight will be recorded to ascertain body mass indices. Dyslipidemia will be defined as total cholesterol ≥240 mg/dL, serum triglycerides ≥150 mg/dL, high‐density lipoprotein‐cholesterol <40 mg/dL in men or <50 mg/dL in women, or diagnosis/treatment of dyslipidemia. Diabetes mellitus (DM) will be defined as fasting plasma glucose >126 mg/dL, glycated hemoglobin (HbA1c) ≥6.5%, or diagnosis/treatment for DM. Prediabetes will be defined as fasting plasma glucose between 100 and 125 mg/dL, HbA1c between 5.7% and 6.4%, or diagnosis/treatment for prediabetes. Presence or absence of diabetes along with current treatment (diet, insulin, or oral medications) will be recorded. Chronic kidney disease will be defined as an estimated glomerular filtration rate less than 60 mL/min/m^2^ prior to admission or a diagnosis of chronic kidney disease or dialysis. Peripheral artery disease will be defined as history of peripheral artery revascularization or a diagnosis of peripheral artery disease confirmed with an ankle‐brachial index <0.90 or lower‐extremity artery stenosis ≥50%. History of smoking will be defined as current (tobacco products used within the last month), former, or never. Emerging risk factors such as hookah smoking, cannabis and cocaine addiction, psychosocial stress, gym supplements consumption is being studied. History of physical activity and possibly therapies like yoga will also be collected.

### 2.6 Medications

Information on baseline, in‐hospital and discharge medications will be prospectively collected from the patient. Use of guideline‐directed medical therapy for ACS will be specifically queried. For antiplatelet therapy, dose of aspirin and type of P2Y_12_ receptor inhibitors will be recorded. For lipid‐lowering medications, use of statins (including intensity), ezetimibe, and proprotein convertase subtilisin/kexin type 9 monoclonal antibodies will be collected. Among patients with known DM, information will also be recorded regarding the use of glucose‐lowering therapies including metformin, sulfonylurea, thiazolidinedione's, glucagon‐like peptide 1 receptor agonists, sodium‐glucose cotransporter 2 inhibitors, dipeptidyl peptidase‐4 inhibitors, insulin, and duration of DM.

### 2.7 Hospital course

On presentation, data on duration from onset of symptoms to presentation and PCI, time of first medical contact and the PCI timing will be recorded. Each hospitalization records will include data on diagnostic testing, laboratory tests, reperfusion strategies, in‐hospital clinical events of interest, length of stay, and discharge status. Information on pharmaco‐invasive therapies will be tabulated as well. In‐hospital clinical events of interest includes complications related to STEMI, such as sudden cardiac death, cardiac arrest, episodes of sustained ventricular tachycardia or ventricular fibrillation, cardiogenic shock, heart failure, mechanical complications of MI such as papillary muscle rupture, severe mitral regurgitation, interventricular septum rupture, ventricular septal defect, cardiac tamponade, or recurrent MI. Furthermore, we will ascertain clinical events related to PCI for STEMI such as access‐site complications, stent thrombosis, bleeding, stroke, and contrast‐induced nephropathy. Clinical endpoints will be assessed and recorded by licensed physicians in the department of cardiology. The collections and details will be supervised on daily basis by the core team before the entry into the system.

### 2.8 Diagnostic evaluation

Medical records will be reviewed to ascertain results of laboratory tests performed during index hospitalization. All study participants will undergo the following laboratory tests on presentation with STEMI: complete blood count, electrolytes, renal function, lipid panel (total cholesterol, low‐density lipoprotein‐cholesterol, triglycerides, and high‐density lipoprotein‐cholesterol, glycosylated hemoglobin, lipoprotein (a), high‐sensitivity C‐reactive protein, and interlukin‐6 levels. Brain natriuretic peptide and cardiac troponin will not be collected initially. However, plasma samples are being collected and stored at −80°C to facilitate future biomarker analysis for all study participants.

Medical records will also be reviewed to document findings on presentation electrocardiograms, invasive coronary angiography, and transthoracic echocardiograms.

### 2.9 Follow‐up

All study participants will be followed at 30 days, 1 year, 3 years, 5 years, and 10‐years after index hospitalization. The follow‐up will be conducted prospectively through in‐clinic follow‐up and through telephone calls. During follow‐up, the vital status, adherence to guideline‐directed medical therapy, and occurrence of cardiovascular events will be assessed. The compliance to drug therapy will be measured on the basis of history from the patient and immediate caregiver at the time of follow‐up. In situations where patients are lost to follow‐up, the vital status will be determined by contacting the designated family member or healthcare provider.

### 2.10 Data management

All study‐related patient data will be stored on a secured password‐protected IOS and Android application software with capabilities for data to be exported into de‐identified encrypted data files for statistical analysis.

### 2.11 Statistical analysis

Continuous variables will be reported as means or medians and compared with *t* tests, Wilcoxon rank‐sum, or analysis of variance, as appropriate. Categorical variables will be reported as proportions and frequencies and will be compared with χ^2^ or Fisher exact tests. Ordinal variables will be compared with a trend test. Multivariable adjusted Cox proportional hazards modeling will be performed for time‐to‐event analysis. Risk factor profiles, clinical characteristics, and outcomes will be analyzed across subgroups stratified by age (<50 vs ≥50 years or age < 35 vs ≥35 years), gender (male vs female), 2013 ACC/AHA cardiovascular risk categories, Thrombolysis in Myocardial Infarction (TIMI)/Global Registry of Acute Coronary Events (GRACE) risk score for secondary prevention, DM, sex, family history of premature CAD, smoking status, history of substance abuse, baseline levels of LDL‐C, triglycerides, lipoprotein (a), hsCRP, IL‐6, glycated hemoglobin, severity of CAD on coronary angiography, left ventricular ejection fraction, and achieved levels of LDL‐C and hsCRP at 1‐month after index presentation.^23^, ^24^, ^25^, ^26^ Multivariable analysis will be performed to identify predictors of subsequent cardiovascular events; the predictors will be used to construct a clinical risk score for prognostication and risk stratification of patients presenting with STEMI in India. All analysis will be performed on de‐identified data. All analysis was performed using SAS 9.4 (Cary, North Carolina).

## 3 RESULTS

We plan to prospectively collect data for approximately 3500 patients who meet our inclusion criteria. From 1 January to 6 March 2019, we have collected data for a total of 558 patients. The baseline characteristics of this initial cohort are detailed in Table 1. The median age was 55 years (interquartile range 45‐61 years), 14% were women, and 34% were younger than 50 years of age. At baseline, 29% had hypertension, 23% diabetes mellitus, 5% hyperlipidemia, 60% were current smokers, and 14% had prior MI. At the time of hospital admission, 17% were on aspirin, 16% on statins, and 13% on β‐blockers. A total of 45% of patients presented to the emergency department more than 1 hour after symptom onset.

**Table 1:** Baseline characteristics of patients currently enrolled in the NORIN STEMI registry (1 January 2019 to 6 March 2019)


**All (N = 558)**	
Age (years), median (IQR)	55 (45‐61)
<50 years, N (%)	191 (34)
<40 years, N (%)	41 (7)
Female sex, N (%)	77 (14)
**Medical history**, **N (%)**	
Diabetes mellitus	129 (23)
Hypertension	188 (34)
Heart failure	9 (2)
Hyperlipidemia	26 (5)
Prior cerebrovascular accident	8 (1)
Myocardial Infarction	80 (15)
Tobacco use	
Never	169 (31)
Former	47 (9)
Current	328 (60)
**Socioeconomic factors**, **N (%)**	
Education	
College graduate	50 (9)
High‐school	108 (19)
Middle school	124 (22)
Illiterate	276 (49)
Diet‐vegetarian	226 (41)
Income strata	
Upper middle	17 (3)
Lower middle	90 (16)
Upper lower	138 (25)
Lower	310 (56)
Alcohol	
Never	362 (65)
Former	54 (10)
Current	140 (25)
**Medications at baseline**, **N (%)**	
Aspirin	93 (17)
Statin	87 (16)
Beta blocker	73 (13)
**Clinical presentation**, **N (%)**	
Time since symptom onset (h)	
<1	307 (55)
1‐3	78 (14)
3‐12	85 (15)
12‐24	29 (5)
≥24	58 (11)
**Laboratory data**	
Serum creatinine (mean ± SD)	1.05 ± 0.42
Hemoglobin (mean ± SD)	13.4 ± 6.0

Abbreviation: IQR, interquartile range.

## 4 DISCUSSION

Clinical practice guidelines, routinely used for the management of STEMI in North America and Europe are based on evidence from high‐income countries that are not representative of patient profiles, practice patterns, healthcare infrastructure, and socioeconomic disparities in India.^27^, ^28^ As there are limited contemporary data of STEMI patients in India, a comprehensive registry will be a major step in understanding the disease burden in India. The NORIN STEMI registry will provide prospective data regarding the prevalence of traditional and emerging risk factors, clinical characteristics, adherence to evidence‐based therapies, and outcomes that may provide us a reflection of the current status of STEMI care in India. Findings from NORIN STEMI will complement those from previous registries of ACS in India, such as the Kerala ACS Registry, a study of 25 748 ACS (37% STEMI) hospitalizations from 2007 to 2009; CREATE, a study of 20 937 patients with ACS (61% STEMI) from 2001 to 2005; and the OASIS registry 1 and 2, a study of approximately 5000 patients with non‐ST‐segment elevation ACS from 1999 to 2000.^13^, ^16^, ^17^, ^18^, ^29^, ^30^, ^31^ This will also provide contemporary data for future initiatives such as the Tamil Nadu STEMI program to devise strategies to improve STEMI care in India.^31^, ^32^ Among patients with STEMI, these registries have consistently shown increasing incidence of STEMI, significant delays in receiving reperfusion therapy with thrombolysis or primary PCI, limited access to hospitals with primary PCI capability, and underutilization of evidence‐based medical therapies.

Furthermore, the NORIN STEMI registry has several unique strengths that will further our understanding of STEMI in India. First, this study offers a an updated perspective on the epidemiology and management of STEMI in India. There is sparse data investigating patients hospitalized with ACS in India in the last decade.^29^, ^33^ Since the last decade has witnessed significant scientific advances in the management of STEMI and since risk factor profiles in India continue to evolve rapidly, it is essential to study a population that represents current practice. Second, the participating institutions provide largely free of cost care to a very diverse population with regards to socioeconomic status, education, and coverage area (largely rural/semi‐urban Indian states). Thus, contrary to previous registries which included patients from a specific region (eg, Kerala ACS registry), our findings will be generalizable to a large portion of the country. Third, we plan to measure novel biomarkers in patients enrolled in this study and are actively storing plasma samples for future biomarker analysis. Evaluation of biomarkers in this high‐risk population will help us discover novel strategies for risk stratification and prognostication for patients with STEMI in India. Finally, unlike previous studies which have only assessed in‐hospital outcomes, we plan to examine both short‐ and long‐term clinical outcomes to follow longitudinally the course of patients after index hospitalization with STEMI.

Considering that prior studies from India have suggested an increasing incidence of STEMI in young individuals, findings from this analysis may help discover novel risk factors that predispose to STEMI at an early age in India. In a resource‐limited country such as India, secondary preventive risk stratification approaches may help identify high‐risk patients post‐STEMI who may derive more benefit from allocation of additional resources (ie, more frequent follow‐up or access to community health workers).

### 4.1 Study limitations

Our study has a few limitations. First, this registry is currently limited to two large academic hospitals in New Delhi; thus, the results may not be generalizable to other practice settings and to other parts of India. However, we plan to include more centers in the future. Second, even though we plan to use multivariable adjustment to account for confounding factors, residual confounding from unmeasured confounders may remain. Third, there is scope of inadequate capture in clinical endpoint reporting due to loss to follow‐up. To minimize this, patients will be followed up by telephone by the same physicians who recorded their details and will be called for regular follow‐up in the outpatient setting.

## 5 CONCLUSION

The NORIN STEMI registry aims to further the understanding of the current burden of STEMI in North India. The findings from this study will provide information regarding contemporary risk factors, clinical profiles, practice patterns, and outcomes of STEMI in India to identify opportunities to improve their outcomes.

## CONFLICT OF INTEREST

Dr. Qamar is supported by the NHLBI T32 postdoctoral training grant (T32HL007604) and the American Heart Association Strategically Focused Research Network in Vascular Disease grant (18SFRN3390085 & 18SFRN33960262), reports receiving grant support through Brigham and Women's Hospital from Daiichi‐Sankyo, fees for educational activities from American College of Cardiology, Society for Cardiovascular Angiography and Interventions, Society for Vascular Medicine, Janssen Pharmaceuticals, Pfizer, Medscape, and Clinical Exercise Physiology Association. Dr. Vaduganathan is supported by the KL2/Catalyst Medical Research Investigator Training award from Harvard Catalyst (NIH/NCATS Award UL 1TR002541), serves on advisory boards for Amgen, AstraZeneca, Baxter Healthcare, Bayer AG, and Boehringer Ingelheim, and participates on clinical endpoint committees for studies sponsored by Novartis and the NIH. Dr. Deepak L. Bhatt discloses the following relationships—Advisory Board: Cardax, Cereno Scientific, Elsevier Practice Update Cardiology, Medscape Cardiology, PhaseBio, Regado Biosciences; Board of Directors: Boston VA Research Institute, Society of Cardiovascular Patient Care, TobeSoft; Chair: American Heart Association Quality Oversight Committee; Data Monitoring Committees: Baim Institute for Clinical Research (formerly Harvard Clinical Research Institute, for the PORTICO trial, funded by St. Jude Medical, now Abbott), Cleveland Clinic (including for the ExCEED trial, funded by Edwards), Duke Clinical Research Institute, Mayo Clinic, Mount Sinai School of Medicine (for the ENVISAGE trial, funded by Daiichi Sankyo), Population Health Research Institute; Honoraria: American College of Cardiology (Senior Associate Editor, Clinical Trials and News, http://acc.org; Vice‐Chair, ACC Accreditation Committee), Baim Institute for Clinical Research (formerly Harvard Clinical Research Institute; RE‐DUAL PCI clinical trial steering committee funded by Boehringer Ingelheim; AEGIS‐II executive committee funded by CSL Behring), Belvoir Publications (Editor in Chief, Harvard Heart Letter), Duke Clinical Research Institute (clinical trial steering committees), HMP Global (Editor in Chief, Journal of Invasive Cardiology), Journal of the American College of Cardiology (Guest Editor; Associate Editor), Medtelligence/ReachMD (CME steering committees), Population Health Research Institute (for the COMPASS operations committee, publications committee, steering committee, and USA national co‐leader, funded by Bayer), Slack Publications (Chief Medical Editor, Cardiology Today's Intervention), Society of Cardiovascular Patient Care (Secretary/Treasurer), WebMD (CME steering committees); Other: Clinical Cardiology (Deputy Editor), NCDR‐ACTION Registry Steering Committee (Chair), VA CART Research and Publications Committee (Chair); Research Funding: Abbott, Amarin, Amgen, AstraZeneca, Bayer, Boehringer Ingelheim, Bristol‐Myers Squibb, Chiesi, CSL Behring, Eisai, Ethicon, Forest Laboratories, Idorsia, Ironwood, Ischemix, Lilly, Medtronic, PhaseBio, Pfizer, Regeneron, Roche, Sanofi Aventis, Synaptic, The Medicines Company; Royalties: Elsevier (Editor, Cardiovascular Intervention: A Companion to Braunwald's Heart Disease); Site Co‐Investigator: Biotronik, Boston Scientific, St. Jude Medical (now Abbott), Svelte; Trustee: American College of Cardiology; Unfunded Research: FlowCo, Fractyl, Merck, Novo Nordisk, PLx Pharma, Takeda. The remaining authors have no conflicts of interest to disclose.

## Supporting information


**Appendix S1.** Supporting Information.
