# Supplementary material for: Design and rationale of the North Indian ST‐Segment Elevation Myocardial Infarction Registry: A prospective cohort study
Source: Clin Cardiol. 2019 Oct 8;42(12):1140–6. doi: 10.1002/clc.23278 (PMC6906983; doi:10.1002/clc.23278)
Supplement: Supplementary file 1 — Appendix S1. Supporting Information. [file CLC-42-1140-s001.docx]

| **Demographic Parameters** |
| --- |
| Name |
| Gender |
| Caregiver details |
| Unique identification number |
| Education |
| Occupation |
| Diet |
| Socioeconomic status |
| Insurance status |
| **History and Risk Factors** |
| Family history of coronary artery disease |
| History of risk factors such as – hypertension, diabetes, atrial fibrillation, prior revascularization, hyperlipidemia |
| Psychosocial history |
| History of substance abuse |
| Mobility Status |
| Medication History/Home Medications |
| **Symptomatology and Initial Treatment** |
| Initial symptoms |
| Exact timeline of symptoms and initial medical contact |
| Nature of First medical contact |
| Causes of delay (if any) |
| Nature of initial treatment given (Thrombolysis or Invasive Angiography) and place of treatment (PCI or Non-PCI center) |
| **Examination and Investigation** |
| Vital parameters |
| Basic anthropometry |
| Echocardiogram findings |
| Routine laboratory investigations |
| Medications administered at arrival |
| **Procedure Related Information** |
| Exact timing of the procedure |
| Pharmaco-invasive therapies |
| Reasons for not performing angiography (if not done) |
| Angiography findings |
| Details of complications (if any) |
| Use of aspiration thrombectomy |
| **Discharge** |
| Status at discharge |
| Medications at discharge |
| **Follow up** |
| Follow-up events: death, readmission, stroke etc. |
| Reasons for unscheduled visits (if any) |
| Regular follow up for events, medication compliance, smoking status |

Appendix Table 1. Brief summary of measured variables in the NORIN-STEMI registry

Abbreviations: PCI: percutaneous coronary intervention

Study Organization:

Principal Investigator: Dr Mohit D Gupta MD DM, Professor, Department of Cardiology, GIPMER.

The site 1 (GIPMER) and Site 2 (JSSH) investigators will be as follows.

| Sites | Site Principal Investigator | Site Co- Principal Investigator |
| --- | --- | --- |
| GIPMER^*^ | Dr Girish MP, MD DM Professor  Department of Cardiology | Dr Ankit Bansal MD DM  Associate Professor  Dr Vishal Batra MD DM  Assistant Professor |
| JSSH^**^ | Dr Puneet Gupta MD DM DNB  Assistant Professor  Department of Cardiology | Dr Gagan Jain MD DM  Associate Professor  Department of Cardiology |

^*^Gobind Ballabh Pant Institute of Post Graduate Medical Education and Research, New Delhi, India

^**^Janakpuri Super speciality Hospital, New Delhi, India

Core Team: Mohit Gupta; Puneet Gupta; Sameer Arora; Arman Qamar

Executive Committee: Mohit Gupta MD DM; Puneet Gupta MD DM; Sameer Arora MD; Arman Qamar MD MPH; Muthiah Vaduganathan MD MPH; Sunil Rao MD; Deepak Bhatt MD MPH; Prashant Kaul MD
